# Changes in the Allostatic Response to Whole-Body Cryotherapy and Static-Stretching Exercises in Chronic Fatigue Syndrome Patients vs. Healthy Individuals

**DOI:** 10.3390/jcm10132795

**Published:** 2021-06-25

**Authors:** Sławomir Kujawski, Anna M. Bach, Joanna Słomko, Derek F. H. Pheby, Modra Murovska, Julia L. Newton, Paweł Zalewski

**Affiliations:** 1 Department of Hygiene, Epidemiology, Ergonomy and Postgraduate Education, Ludwik Rydygier Collegium Medicum in Bydgoszcz Nicolaus Copernicus University in Torun, M. Sklodowskiej-Curie 9, 85-094 Bydgoszcz, Poland; 264063@stud.umk.pl (A.M.B.); jslomko@cm.umk.pl (J.S.); p.zalewski@cm.umk.pl (P.Z.); 2 Society and Health, Buckinghamshire New University, High Wycombe HP11 2JZ, UK; derekpheby@btinternet.com; 3 Institute of Microbiology and Virology, Riga Stradiņš University, LV-1067 Riga, Latvia; Modra.Murovska@rsu.lv; 4 Population Health Sciences Institute, The Medical School, Newcastle University, Framlington Place, Newcastle-upon-Tyne NE2 4HH, UK; julia.newton@newcastle.ac.uk

**Keywords:** cold therapy, physical activity, stretching

## Abstract

This study represents a comparison of the functional interrelation of fatigue and cognitive, cardiovascular and autonomic nervous systems in a group of Chronic Fatigue Syndrome (CFS) patients compared with those in healthy individuals at different stages of analysis: at baseline and after changes induced by whole-body cryotherapy (WBC) combined with a static-stretching (SS) program. The study included 32 patients (Fukuda criteria) and 18 healthy controls. Fatigue, cognitive, cardiovascular and autonomic function and arterial stiffness were measured before and after 10 sessions of WBC with SS. In the patients, a disturbance in homeostasis was observed. The network relationship based on differences before and after intervention showed comparatively higher stress and eccentricity in the CFS group: 50.9 ± 56.1 vs. 6.35 ± 8.72, *p* = 0.002, r = 0.28; and 4.8 ± 0.7 vs. 2.4 ± 1, *p* < 0.001, r = 0.46, respectively. Before and after intervention, in the CFS group increased fatigue was related to baroreceptor function, and baroreceptor function was in turn related to aortic stiffness, but no such relationships were observed in the control group. Differences in the network structure underlying the interrelation among the four measured criteria were observed in both groups, before the intervention and after ten sessions of whole cryotherapy with a static stretching exercise.

## 1. Introduction

Whole body cryotherapy (WBC) has been proven to be a safe and efficient form of physiotherapy in both sports medicine (e.g., skeletal disorders [1,2] and acute and chronic injuries [3]) and clinical settings (e.g., fibromyalgia [4] and neurological disorders [4]). WBC activates physiological mechanisms that maintain a constant core temperature. Acute exposure to ambient cryotherapeutic temperatures is an extremely stressful stimulant that induces rapid and short-term regulatory mechanisms predominantly associated with the cardiovascular and autonomic nervous systems [5]. In addition, WBC programs have been shown to improve immediate recall and orientation in older participants with mild cognitive impairment and reduce fatigue in multiple sclerosis (MS) patients [6,7]. Pawik et al. showed that WBC with physical training was more effective than WBC alone in reducing depressive symptoms and improving functional status in MS patients [8]. 

The influence of flexibility (stretching) exercises on cardiovascular and autonomic nervous system responses has been explored [9,10,11,12]. The American College of Sports Medicine (ACSM) describes flexibility as one of the most important components of physical fitness [13]. Stretching is a non-pharmacological, low-intensity activity that can improve vascular function for those with cardiovascular disorders and chronic fatigue disorders and elicits a lower metabolic demand compared to moderate or vigorous aerobic exercise [14,15,16]. Planned exercise therapy to strengthen weakened muscle or to increase joint flexibility is often included as part of a treatment program for patients with chronic fatigue and chronic fatigue syndrome (CFS), a common condition that remains difficult to diagnose and manage. Some of the current challenges include an absence of diagnostic markers and differing diagnostic criteria [17]. Post-exertional malaise (PEM) can be induced by physical exercise in many CFS patients [18], so pacing is proposed as a method for managing energy [19]. A stretching program characterized by relatively low intensity aerobic or resistance exercise might be more effective in avoiding PEM. In their review Kruse and Scheuermann described the unequivocal influence of acute and long-term stretching exercise responses on aortic stiffness, cardiovascular and autonomic nervous system functioning, specifically an increase in parasympathetic and decrease in sympathetic activity [20]. Therefore, it might be applied in CFS patients to treat secondary symptoms. Autonomic nervous system (ANS) dysfunction is widely implicated as potentially underpinning CFS pathomechanisms [21]. Słomko et al. showed that patients with a sympathetic profile (i.e., higher sympathetic activity at rest measured objectively) were characterized by markedly low fatigue and subjective autonomic function, high objective autonomic function and markedly high aortic stiffness. The parasympathetic profile (higher parasympathetic activity at rest) showed a more severe disease with average subjective autonomic function, low objective autonomic function levels and high arterial stiffness [22].

The 2015 Institute of Medicine (IOM) report highlighted an increasing body of literature citing cognitive impairment as a key symptom in people suffering from CFS [23]. One study noted a prevalence of 80% or more in brain fog/confusion, short-term memory problems, trouble concentrating, slow thinking, difficulty understanding/thinking clearly and difficulty retaining information [24]. Between 50 and 85% of CFS patients noted subjective cognitive impairment [25]. So-called brain fog includes a general slowdown in response speed with tasks that require simple and complex information processing as well as the ability to focus on a task [25]. Studies also confirmed that cognitive disturbances are restricted to a decrease in basic processing speed, not to severity of depression [26,27]. Thayer and Lane promoted a neurovisceral integration model and underlined the contribution of vagal heart rate variability (HRV) in the control of physiological, emotional and cognitive processes. Low HRV is a risk factor for pathophysiology and psychopathology [28]. Further studies have confirmed a correlation between cognitive and autonomic nervous system function [29,30] and between autonomic nervous system function and brain pathologies [31]. 

In summary, WBC with a stretching program might improve autonomic nervous system function [5,6,7,20] A previous CFS study using a network analysis approach showed evidence of disturbance glucose metabolism and in homeostasis in the autonomic nervous and cardiovascular systems [32]. Thus, it seems that autonomic nervous system function, cognitive function and aortic stiffness could be related to fatigue in CFS. 

Allostasis is defined as the “process of maintaining homeostasis through the adaptive change of the organism’s internal environment to meet perceived and anticipated demands” [33], and in the current study allostasis in CFS patients was compared to healthy controls, before and in response to an intervention consisting of WBC and SS. The aim of this study was to explore differences in the relationship among fatigue, autonomic nervous system function, aortic stiffness and cognitive function in CFS compared to controls, both before and in response to whole body cryotherapy and static stretching. 

## 2. Materials and Methods

### 2.1. Patients

Out of 250 individuals who identified themselves as fatigued, 32 patients with chronic fatigue syndrome according to the Fukuda criteria were selected [34]. The inclusion criteria for the study were men and women between 25 and 65 years, fatigued due to unknown causes lasting for more than 6 months and at least four of the following additional symptoms: malaise after exertion, impaired memory or concentration, headache, unrefreshing sleep, tender lymph nodes (cervical or axillary), sore throat, or muscle or joint pain. The exclusion criterion was the presence of an illness that might trigger chronic fatigue (e.g., a cardiovascular or autoimmune disease or a psychosocial cause). Patients could participate in this study if they had been referred by a general practitioner, neurologist or psychiatrist. A pre-test health assessment was carried out that included basic psychiatric and neurological examinations. A consultant confirmed conformity with the inclusion and exclusion criteria and determined whether an extensive physical examination or laboratory research tests had been performed to exclude any underlying illness.

**Figure 1:**
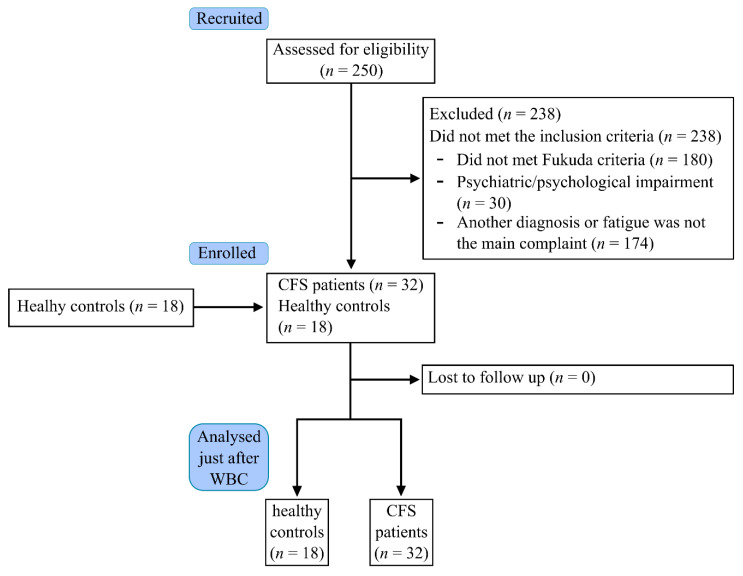
Flow chart of the study.

Eighteen healthy individuals were also recruited from the community as a control group (HC). Thus, 32 CFSs and 18 HCs participated (Figure 1).

### 2.2. Intervention—Whole Body Cryotherapy (WBC) with a Static Stretching Exercise

The WBC procedures were performed in a liquid nitrogen cryochamber consisting of two compartments: an antechamber and a chamber proper, which were connected by a door. In the trial, the temperature in the antechamber was set at −60 °C, whereas in the proper chamber, it reached −120 °C.

**Figure 2:**
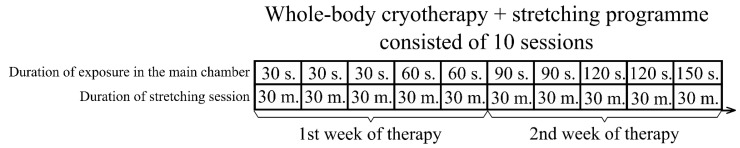
Whole-body cryotherapy with stretching intervention timeline.

The participants were treated using a cycle of 10 visits over a period of 12 days (from Monday to Friday, one session per day). They were subjected to the effects of extremely low temperature in a WBC chamber for 0.5–2.5 min depending upon the day of therapy. Exposure time was incremental: 1–3 days for 0.5 min, 4–5 days for 1 min, 6–7 days for 1.5 min, 8–9 days for 2 min and 10 days for 2.5 min (Figure 2). 

To protect the most sensitive body areas, all patients entering the rooms were dressed in swimsuits, a face mask, cotton socks, slippers, gloves, ear-protectors and wooden shoes. All jewelry, glasses and contact lenses were removed before entry. During the WBC procedure, participants in the chamber walked round without touching each other.

**Figure 3:**
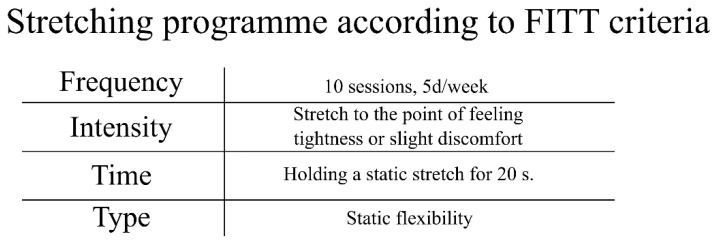
Whole-body cryotherapy with stretching frequency, intensity, time and type (FITT) according to ACSM criteria [29].

Immediately after leaving, they underwent a static stretching exercise session (Figure 3). According to ACSM recommendations for flexibility [13], our program included static stretching of the major muscle tendon units of the shoulder girdle, chest, neck, trunk, lower back, hips, posterior and anterior legs and ankles from small to large muscle groups. Patients exercised in a lying position, gradually moving to sitting and standing. Each stretch was held to the point of slight discomfort (not pain) for a period of 20 s per muscle group followed by a 10 s passive rest period in a neutral position [35]. All sessions of WBC and physical exercise were carried out under the supervision of a medical doctor and experienced physiotherapists.

### 2.3. Measures

The clinical examination was performed in the chronobiology laboratory (temperature 22 °C, humidity 60%, windowless and sound-insulated) at approximately the same time of day.

#### 2.3.1. Fatigue Measurements

The Chalder Fatigue Questionnaire (CFQ), the Fatigue Severity Scale (FSS) and the Fatigue Impact Scale (FIS) were used to evaluate severity.

The Chalder Fatigue Questionnaire consisted of 11 questions separated into two dimensions of fatigue—physical (items 1–7) and mental (items 8–11). This scale, scored in “Likert” style, asked individuals to rate the severity of fatigue as 0, 1, 2 or 3 for a total score of 0–33. The mean “Likert” score was 24.4 (SD 5.8) and 14.2 (SD 4.6) for physical and mental fatigue, respectively [36]. 

The Fatigue Severity Scale (FSS) included nine statements that rated the severity of fatigue symptoms on a scale of 1 to 7, based on how accurately it reflected their condition during the previous week. A total score of 36 or more suggested fatigue [37]. 

The Fatigue Impact Scale (FIS) had 40 items, scored on a 5-point Likert scale (0–4), providing a continuous scale of 0–160 with a higher score indicating greater fatigue [38].

#### 2.3.2. Assessment of Cognitive Function

The Trail Making Test was used to evaluate various neuropsychological skills including the executive functioning domain: working memory, visuospatial skills and task switching [39]. The Trial Making Test Part A (TMT A) required the connection of dots with numbers from 1 to 25 in sequential order to determine visual search and motor speed skills. Part B of the test (TMT B) was used to test cognitive flexibility and executive control, requiring the subjects to connect a number with a letter and then place them in ascending order (e.g., 1-A, 2-B.) [40,41,42,43].

In the coding test––an indicator of attention, working memory, motor speed, scanning, associative learning and executive functions––the subjects had to write down the corresponding symbol to every digit in a consecutive manner as fast as possible. The result recorded the number of digits left to be encoded after 60 and 120 s. If a subject was not able to finish the task within 120 s, the task was interrupted [39,44].

#### 2.3.3. Objective Assessment of Autonomic and Cardiovascular Function

The hemodynamic and left ventricular function parameters recorded were heart rate (HR), mean blood pressure (mBP), stroke index (SI), cardiac index (CI), total peripheral resistance index (TPRI), left ventricular ejection time (LVET), pre-ejection period (PEP), left ventricular work index (LVWI) and the Heather index (HI). Autonomic parameters included the ratio of low-frequency (LF) to high-frequency (HF) diastolic blood pressure of the r to r interval (LF/HF). Baroreceptor sensitivity was measured in a beat-to beat manner (Total BEI). Parameters were measured while supine after 5 min waiting for parameter normalization with a dedicated Task Force Monitor (TFM, CNSystems, Medizintechnik, Graz, Austria). The main area of TFM application was as an automated beat-to-beat analysis of an electrocardiogram (ECG), impedance cardiography (ICG) and oscillometric and non-invasive continuous blood pressure measurements (oscBP, contBP). TFM automatically provided a power spectral analysis for HRV and BPV conducted using the adaptive autoregressive model proposed by Bianchi et al. [45,46,47].

#### 2.3.4. Arterial Stiffness Measurement

Vascular stiffness measurements were performed by the Arteriograph (TensioMed Software v.1.9.9.2; TensioMed, Budapest, Hungary), which determined pulse wave velocity (PWV) and augmentation index (Aix) as well as the aortic systolic blood pressure (sBP aortic) by analysing the oscillometric pressure curves registered on the upper arm [48,49]. 

#### 2.3.5. Body Composition Analysis

To measure body composition changes, a multifrequency, bioelectrical impedance analyzer (Tanita MC-180MA Body Composition Analyzer, Tanita, Manchester, UK) was applied. All subjects were given a “normal” proprietary algorithm for the impedance measurement.

### 2.4. Statistical Analysis

Mean and standard deviation (±SD) values were presented, and the Shapiro–Wilk test and histograms were used to test the assumption of normality. Levene’s test was used to test the assumption of homogeneity of variance, while. Mann–Whitney U or independent *t*-tests were used to examine intergroup differences depending on meeting certain assumptions. Effect size (r) was calculated for significance between group comparisons [50]. Spearman’s rank correlation coefficient was used (Spearman’s ρ and *p*-value). Parametric methods were not used because the majority of variables did not meet assumptions. The network analysis was performed using Cytoscape software version 3.8.1 [51]. The variables were grouped according to categories illustrated by node colour: cognitive function (green), fatigue scales (blue), vascular function (magenta), cardiac muscle function (red) and autonomic nervous system function (brown). The size of the dots next to the variable name is continuously related to the number of statistically significant correlation coefficients with other variables (assuming alpha = 0.05). Statistically significant correlations were only presented in form of edges. The colours of the edges denoted the direction of correlation: blue indicated a negative while red indicated a positive correlation. Edge width and intensity of colour denoted the strength of the relationship. A Prefuse Force Directed Layout algorithm was applied based on a non-negative strength correlation. A Cytoscape software tool was used to analyse the networks. Based on a dyshomeostasis study [32], the values for the number of edges per node, topological coefficient (relative measure for the extent of node sharing with neighbors) neighbourhood connectivity (the average connectivity among all neighbours of a node), stress (the number of shortest paths through each node), eccentricity (maximum distance from v to any other node) and clustering coefficient (the degree to which nodes in a graph tend to cluster together) were compared using the Mann–Whitney U test. Differences between groups (CFS vs. control) before intervention and in delta value (before and after WBC and SS) were analysed.

## 3. Results

### 3.1. Network Relationship before Intervention

Data obtained from 32 patients (26 females) with CFS and 18 control participants (10 females) were included. Baseline characteristics were similar between the two groups. As shown in Table 1, differences were found in age, body composition and BMI.

**Figure 4:**
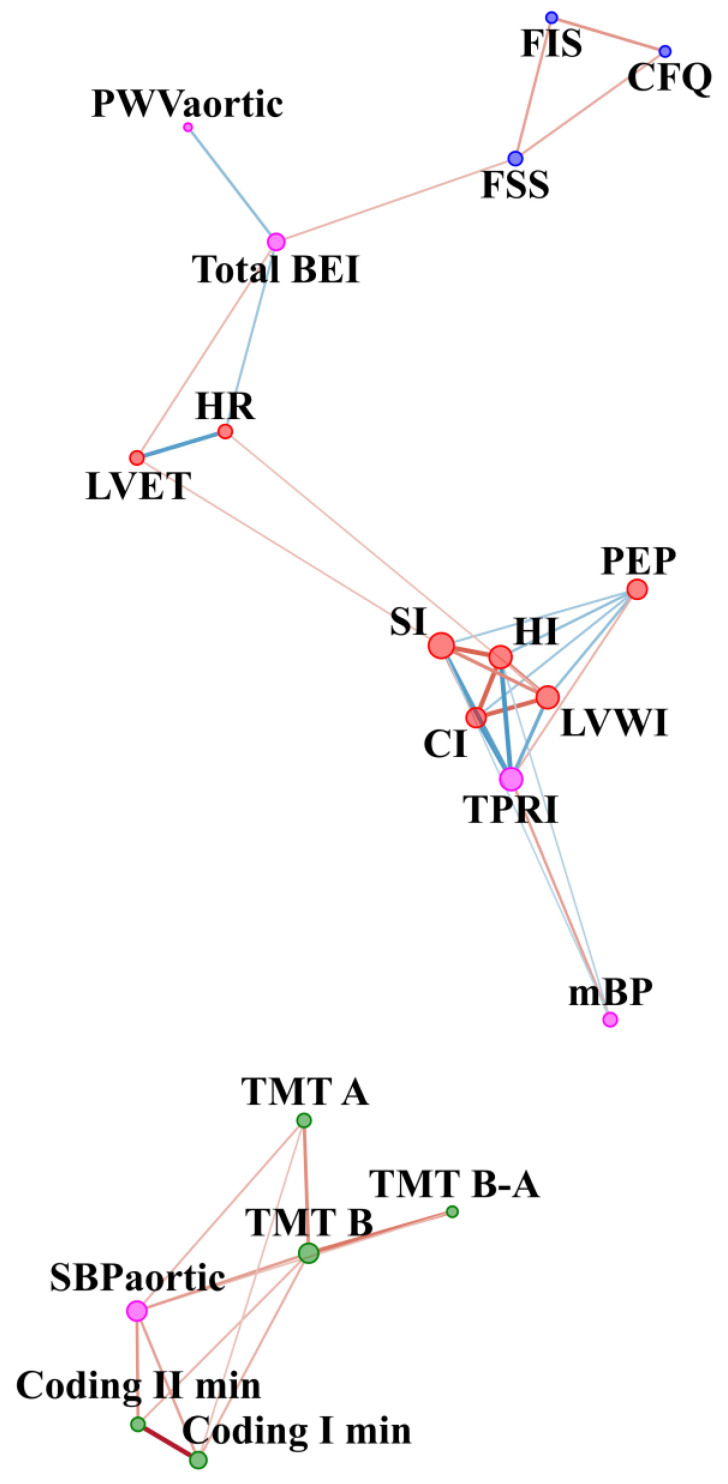
Network analysis in CFS patients before WBC and SS therapy. The variables were grouped according to categories illustrated by colour of nodes: cognitive function (green), fatigue scales (blue), vascular system function (magenta) and cardiac muscle function (red). The size of the dots next to the variable names is continuously related to the number of statistically significant correlation coefficients with other variables. The colour of edges denotes the sign of correlation: blue indicate negative, while red indicates positive correlation. Edge width and intensity of colour denote strength of relationship. CFQ—Chronic Fatigue Scale, FSS—Fatigue Severity Scale, FIS—Fatigue Impact Scale, HR—Hear Rate, SI—Stroke Index, HI—Heather index, PEP—Pre-ejection period, LVET—Left Ventricular Ejection Time, CI—Cardiac Index, LVWI—Left Ventricle Work Index, TPRI—Total Peripheral Resistance Index, mBP—Mean Blood Pressure, PWVaortic—Aortic Pulse Wave Velocity, SBPaortic—central systolic blood pressure, Total BEI—baroreceptor effectiveness index, TMT A—Trial Making Test part A, TMT B—Trial Making Test part B, TMT B-A—Difference in result between TMT B and TMT A, Coding I—number of words left after first minute of coding test; Coding II—number of words left after second minute of coding test.

**Figure 5:**
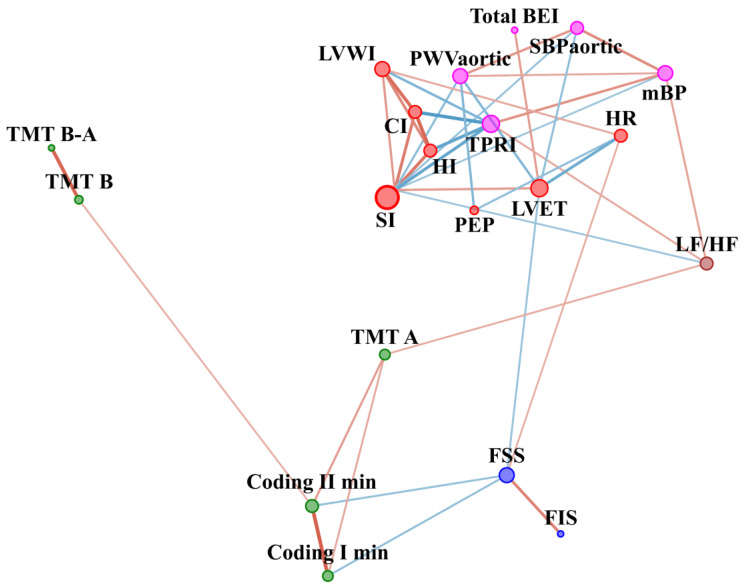
Network analysis in the control group before WBC and SS therapy. The variables were grouped according to categories illustrated by colour of nodes: cognitive function (green), fatigue scales (blue), vascular system function (magenta) cardiac muscle function (red), autonomic nervous system function (brown). The size of the dots next to the variable names is continuously related to the number of statistically significant correlation coefficients with other variables. The colour of the edges denotes the sign of correlation: blue indicates negative, while red indicates positive. Edge width and intensity of colour denote strength of relationship. CFQ—Chronic Fatigue Scale, FSS—Fatigue Severity Scale, FIS—Fatigue Impact Scale, HR—Hear Rate, SI—Stroke Index, HI—Heather index, PEP—Pre-ejection period, LVET—Left Ventricular Ejection Time, CI—Cardiac Index, LVWI—Left Ventricle Work Index, TPRI—Total Peripheral Resistance Index, mBP—Mean Blood Pressure, PWVaortic—Aortic Pulse Wave Velocity, SBPaortic—central systolic blood pressure, Total BEI—baroreceptor effectiveness index, LF/HF—sympathovagal balance, TMT A—Trial Making Test part A, TMT B—Trial Making Test part B, TMT B-A—Difference in result between TMT B and TMT A, Coding I—number of words left after first minute of coding test; Coding II—number of words left after second minute of coding test.

The comparison of CFS patients with the controls before WBC and SS in respect of other parameters is included in Table A1 (Appendix A). In the CFS group before WBC, cognitive function was positively related to the central systolic blood pressure, namely, the higher the sBP aortic the worse the cognitive function (Figure 4). In contrast, sBPaortic had no significant edges in the control group (Figure 5). In the CFS group, fatigue scale results were related to baroreceptor function, and baroreceptor function was related to aortic stiffness, but no such edges were observed in the control group. On the other hand, in the control group, fatigue scales results were related to cognitive function, but no such connection was observed in the CFS patients. In addition, the LF/HF node was not related to other nodes in the CFS group. In contrast, LF/HF contained 3 edges connected with parameters related to blood pressure regulation and cognitive function in the control group.

Before WBC, eccentricity was lower in the CFS network compared to that of controls (3.5 ± 1.4 vs. 4.7 ± 0.8, *p* = 0.01, r = −0.22). Topological and clustering coefficients were higher in the CFS network than for the controls (0.67 ± 0.2 vs. 0.4 ± 0.2, *p* = 0.0004, r = 0.32 and 0.7 ± 0.3 vs. 0.4 ± 0.3, *p* = 0.01, r = 0.23, respectively.

### 3.2. Network Relationships in Difference before and after Intervention (Delta Value)

**Figure 6:**
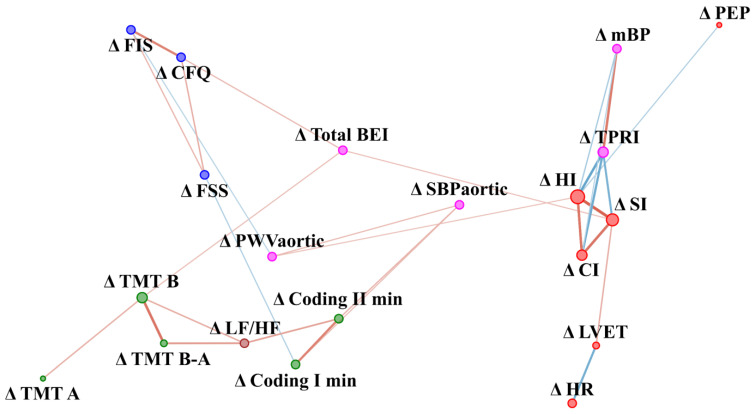
Network analysis of difference in parameters value before and after WBC and SS therapy in CFS patients. The variables were grouped according to categories illustrated by colour of nodes: cognitive function (green), fatigue scales (blue), vascular system function (magenta), cardiac muscle function (red) and autonomic nervous system function (brown). The size of the dots next to the variable names is continuously related to the number of statistically significant correlation coefficients with other variables. Colour of edges denotes sign of correlation: blue indicate negative, while red indicates positive correlation. Edge width and intensity of colour denote the strength of the relationship. Δ (delta) refers to differences in result before and after the WBC and stretching programme. CFQ—Chronic Fatigue Scale, FSS—Fatigue Severity Scale, FIS—Fatigue Impact Scale, HR—Hear Rate, SI—Stroke Index, HI—Heather index, PEP—Pre-ejection period, LVET—Left Ventricular Ejection Time, CI—Cardiac Index, LVWI—Left Ventricle Work Index, TPRI—Total Peripheral Resistance Index, mBP—Mean Blood Pressure, PWVaortic—Aortic Pulse Wave Velocity, SBPaortic—central systolic blood pressure, Total BEI—baroreceptor effectiveness index, LF/HF—sympathovagal balance, TMT A—Trial Making Test part A, TMT B—Trial Making Test part B, TMT B-A—Difference in result between TMT B and TMT A, Coding I—number of words left after first minute of coding test; Coding II—number of words left after second minute of coding test.

**Figure 7:**
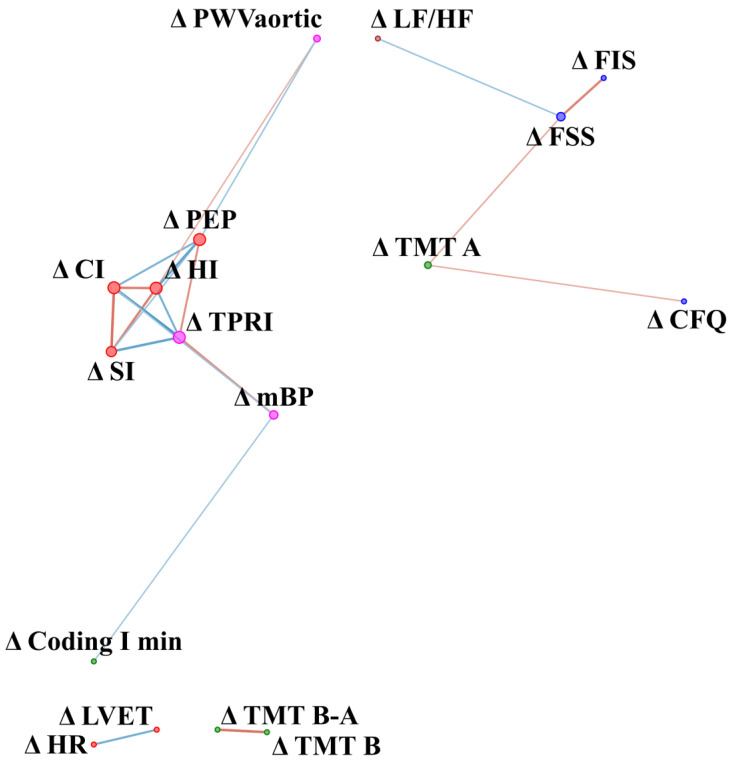
Network analysis of difference in parameters value after-before (delta) WBC and SS therapy in control group. The variables were grouped according to categories illustrated by colour of nodes: cognitive function (green), fatigue scales (blue), vascular system function (magenta, cardiac muscle function (red), autonomic nervous system function (brown). The size of the dots next to the variable names is continuously related to the number of statistically significant correlation coefficients with other variables. Colour of edges denote sign of correlation: blue indicates negative while red indicates positive correlation. Edge width and intensity of colour denote strength of relationship. Δ (delta) refers to difference in result before and after WBC and stretching program. CFQ—Chronic Fatigue Scale, FSS—Fatigue Severity Scale, FIS—Fatigue Impact Scale, HR—Hear Rate, SI—Stroke Index, HI—Heather index, PEP—Pre-ejection period, LVET—Left Ventricular Ejection Time, CI—Cardiac Index, LVWI—Left Ventricle Work Index, TPRI—Total Peripheral Resistance Index, mBP—Mean Blood Pressure, PWVaortic—Aortic Pulse Wave Velocity, SBPaortic—central systolic blood pressure, Total BEI—baroreceptor effectiveness index, LF/HF—sympathovagal balance, TMT A—Trial Making Test part A, TMT B—Trial Making Test part B, TMT B-A—Difference in result between TMT B and TMT A, Coding I—number of words left after first minute of coding test; Coding II—number of words left after second minute of coding test.

Fatigue was not measured after WBC in the control group, so baseline values were included in the analysis. In the CFS group changes (delta) in fatigue scale results in response to WBC were related to changes in baroreceptor function and aortic stiffness, while in the control group fatigue at baseline was related to changes in the autonomic nervous system (Figure 6). In the control group, changes (delta) in TMT A were related negatively to changes in fatigue scales (CFQ and FSS) (Figure 7). Stress and eccentricity of the network were higher in the CFS group compared to control (50.9 ± 56.1 vs. 6.35 ± 8.72, *p* = 0.002, r = 0.28 and 4.8 0.7 vs. 2.4 1, *p* = 0.0000004, r = 0.46, respectively).

## 4. Discussion

A network analysis has been successfully applied in medicine [52] and physiology [53]. Such an approach might facilitate bridging the knowledge gap concerning the interaction between physiological systems [53]. This application of network analysis is considered to be an emerging field, not free from limitations. Ivanov et al. note that there is a need to gather more data and to develop new methods of analysis that could be applied to physiology [53].

### 4.1. General Topography of Networks

In CFS patients, homeostasis disturbance was observed. Our exploratory analysis showed differences in the network structure underlying the relationships between cognitive function, fatigue, cardiovascular and autonomic function in both general topology and amongst specific nodes and their interactions. In part we confirmed the results of a previous study, where a disturbance in autonomic and cardiovascular homeostasis and glucose metabolism were noted in the CFS patients [32]. A similar observation on general topography in comparison to the previous study was noted. Before WBC and stretching, the autonomic network in the CFS patients showed a more uneven distribution of information [32]. In the current study, before WBC and stretching, nodes illustrating cognitive function test results were only connected to nodes in the same category, while the central systolic blood pressure was the only noted connection outside of this group. In comparison, both the current and the previous study found that networks in the control group were distributed in a more regular and balanced fashion. Clark et al. noted that this was in line with theoretical computational models [54,55] and the results of studies based on data from human studies [56]. In the previous study, stroke volume was one of three centres crucial in the CFS group network [32]. Accordingly, in the current study, the stroke index was also a node characterized by higher connectivity compared to other nodes, both before WBC and SS and in the differences between before and after. In the current study, a change in SI in response to WBC and SS was related to changes in baroreceptors function, while no such connection was observed in the control group.

### 4.2. Network before WBC with Static Stretching (SS) Program in CFS vs. Healthy Controls

In the CFS group before WBC, cognitive function was positively related to central systolic blood pressure, namely, the higher the sBP aortic the worse the cognitive function. In contrast, sBPaortic had no significant edges in the control group. Negative correlation between aortic stiffness and cognitive function in the CFS group was in accordance with previous results where higher aortic stiffness was related to worse cognitive function in older people [57]. Increased aortic stiffness could be related to higher flow pulsatility and an excessive pulsatile load on the heart, as well as to microvascular lesions in organs with high metabolic demands, such as the brain [58].

In the CFS group, fatigue levels related to baroreceptor function, and baroreceptor function was, in turn, related to aortic stiffness, while no such edges were observed in the control group. Orthostatic intolerance is one of the hallmark symptoms of CFS [59]. In previous studies, lower ambulatory blood pressure had been observed in CFS patients compared to healthy controls [60] as had disturbance in blood pressure control [61,62]. Baroreceptor reflex sensitivity was disturbed to a greater degree in severe CFS patients than in those with less severity [63]. In a recent study of fibromyalgia patients, fatigue was related to baroreflex dysfunction [64]. 

In addition, in the CFS group, sympathovagal balance was absent in the network, compared to the control group, where it contained 3 edges connecting with parameters related to blood pressure regulation and cognitive function. Observation in the healthy controls group was in line with previous studies exploring the relationship between the autonomic nervous system and cognitive function [30,31]. 

The lack of a significant relationship between fatigue severity and cognitive function was a surprise, but such a relationship was observed in the control group. Brain fog includes a generally slower response speed for tasks that require simple and complex information processing and a lesser ability to focus on a task [26]. Studies also confirm that cognitive disturbance in ME/CFS patients is restricted to a decrease in basic processing speed and is not related to depression severity [27,29]. However, brain fog intensity might presumably not be directly related to fatigue intensity. Further studies should be done to examine the nature of the relationship between these specific symptoms frequently seen in CFS patients.

### 4.3. Dynamics of the Network of WBC with Static Stretching (SS) Program in CFS vs. Healthy Controls

In the CFS group, changes in fatigue in response to WBC and SS were related to changes in baroreceptor function and aortic stiffness, while in the control group fatigue during baseline was related to changes in autonomic parameters. Moreover, changes in cognitive function were related to changes in the LF/HF ratio, namely the sympathovagal balance and to the results from one of the fatigue scales (FIS). Further studies should determine if a decrease in brain fog intensity is related to therapies that improve autonomic function and reduce fatigue. A previous study of multiple sclerosis patients showed that WBC might reduce fatigue [7], while Rymaszewska et al. showed that it might lead to improvement in some domains of cognitive function [6]. Moreover, stretching can reduce fatigue in call-centre workers [65] and in high school students [66]. A stretching training program might induce a variety of physiological adaptations [20]. HR increase in response to stretching is transient: a rapid rise of 4–5 beats/minute was noted and then a return to baseline occurs within several seconds [67,68,69]. Moreover, the authors of a review on stretching effects noted an increase in parasympathetic and a decrease in sympathetic activity after subjects exercised all the large muscle groups, but rather unequivocal effects for aortic stiffness [20]. Presumably, cognitive function improvement might be related to autonomic nervous system changes and fatigue reduction. Further studies should examine the influence of different therapies on brain fog and its underlying mechanism in CFS.

### 4.4. Study Limitations

The same intervention (WBC and SS) was applied to both the CFS and healthy control group. In further studies, the effects of WBC should be compared to SS solely and in combination. In the current study healthy controls were not age-matched to CFS groups. However, no significant differences in age or BMI and body composition were found. One of the study limitations was that the results were not adjusted for multiple corrections. The Fukuda criteria for CFS were used [70,71,72], but consideration might be given to combining Fukuda with others: for instance, the International Consensus Criteria [72]. 

### 4.5. Clinical Implications

Our study was the first to show the effects of whole-body cryotherapy with a static stretching exercise on the network relationship between fatigue, cognitive function, the cardiovascular and autonomic nervous systems in a group of patients with Chronic Fatigue Syndrome. Our exploratory analysis showed that a difference in the network structure was observed both before the intervention and in the difference before and after 10 sessions of WBC with static stretching. Further studies involving different orders of time and dose response to WBC are still required to understand the mechanisms involved in fatigue reduction for it to be safely prescribed and more widely used in CFS patient management. In addition, certain stressors such as physical exercise or cognitive effort might induce PEM in CFS patients [73]. For example, in our previous study based on an aerobic activity program, 35 of 69 patients were unable to tolerate the intervention [74]. In the above study, all the CFS patients who started the intervention completed it. Further studies should consider whether PEM can be induced by any type of stressor or whether it is more related to certain types of activity, such as strenuous physical exercise. In addition, the effects of WBC and a stretching program should be examined with patients experiencing different intensities of symptoms.

## Figures and Tables

**Table 1:** Comparison of CFS vs. controls before WBC and SS.

	CFS (*n* = 32)	Control (*n* = 18)	
Parameter	Mean ± SD	Mean ± SD	*p*-Value
Age (years)	36.72 ± 8.4	38.39	0.50
Body height (cm)	170.4 ± 8.3	172.6 ± 9.8	0.41
Body mass (kg)	72.2 ± 12.7	77.6 ± 20.3	0.27
BMI (kg/m^2^)	24.8 ± 3.6	25.8 ± 5.7	0.4
BMR (kcal)	6477.5 ± 1010.1	7029.1 ± 1624.2	0.15
FatP (%)	27.7 ± 7.5	26.4 ± 7.8	0.57
FatM (%)	20.4 ± 8.0	21.2 ± 10.5	0.75
FFM (kg)	51.8 ± 8.7	56.4 ± 12.9	0.14
VFat (level)	4.6 ± 2.4	5.9 ± 4.1	0.18
BoneM (kg)	2.6 ± 0.4	2.8 ± 0.6	0.16

BMI—body mass index, BMR—basal metabolic rate, FatP—body fat percentage, FatM—body fat mass, FFM—free-fat mass, VFat—Visceral fat level, BoneM—bone mass.

## Data Availability

Individual data is available from the corresponding author S.K. on request.

## Appendix A

**Table A1:** Comparison of CFS vs. controls before WBC and SS.

	CFS (*n* = 32)	Control (*n* = 18)	
Parameter	Mean ± SD	Mean ± SD	*p*-Value
TMT A (s)	23.03 ± 6.2	24.50 ± 7.6	0.5
TMT B (s)	50.19 ± 13.9	59.72 ± 25.0	0.09
TMT B-A (s)	27.16 ± 11.4	35.22 ± 23.7	0.32
Coding 1 min (symbols_to_go)	52.81 ± 9.5	55.17 ± 7.3	0.35
Coding 2 min (symbols_to_go)	12.68 ± 11.5	15.11 ± 11.1	0.48
CFQ (points)	22.06 ± 4.5	12.50 ± 3.3	<0.001
FIS (points)	54.47 ± 24.2	22.72 ± 19.5	<0.001
FSS (points)	45.41 ± 8.7	30.00 ± 11.4	<0.001
PWV aortic (m/s)	8.41 ± 2.1	8.06 ± 1.6	0.89
Aix aortic (%)	28.72 ± 12.4	22.45 ± 11.4	0.08
sBP aortic (mmHg)	135.06 ± 17.8	128.91 ± 19.0	0.25
HR (n/1)	69.98 ± 8.8	75.56 ± 10.5	0.05
mBP (mmHg)	95.80 ± 8.9	96.41 ± 11.0	0.83
SI (mL/m^2^)	54.22 ± 12.6	48.97 ± 10.3	0.14
CI (L/min/m^2^)	3.76 ± 0.9	3.67 ± 0.9	0.71
TPRI (dyne*s*m^2^/cm^5^)	2127.60 ± 666.2	2167.97 ± 579.0	0.59
LVWI (mmHg*L/(min*m^2^))	4.78 ± 1.1	4.68 ± 1.0	0.77
LVET (ms)	316.79 ± 12.0	305.36 ± 16.1	0.01
PEP (ms)	110.07 ± 14.2	104.60 ± 12.7	0.18
HI (1/s²)	0.37 ± 0.1	0.33 ± 0.1	0.27
LF/HF	1.60 ± 1.9	2.48 ± 1.7	0.002
Total BEI (%)	67.28 ± 11.6	65.95 ± 22.8	0.79

CFQ—Chronic Fatigue Scale, FSS—Fatigue Severity Scale, FIS—Fatigue Impact Scale, HR—Hear Rate, SI—Stroke Index, HI—Heather index, PEP—Preejection period, LVET—Left Ventricular Ejection Time, CI—Cardiac Index, LVWI—Left Ventricle Work Index, TPRI—Total Peripheral Resistance Index, mBP—Mean Blood Pressure, PWVaortic—Aortic Pulse Wave Velocity, SBPaortic—central systolic blood pressure, Total BEI—baroreceptor effectiveness index, LF/HF—sympathovagal balance, TMT A—Trial Making Test part A, TMT B—Trial Making Test part B, TMT B-A—Difference in result between TMT B and TMT A, Coding I—min-number of words left after 1st minute of coding test, Coding II—min-number of words left after 2nd minute of coding test.
